# Radioprotective effects of melatonin and thymoquinone on liver, parotid gland, brain, and testis of rats exposed to total body irradiation

**DOI:** 10.55730/1300-0144.5654

**Published:** 2023-02-02

**Authors:** Mehmet KOÇ, Ciğdem Damla DENİZ, Mehmet Akif ERYILMAZ, Yılmaz TEZCAN, Mehmet GÜRBİLEK

**Affiliations:** 1 Department of Radiation Oncology, Faculty of Medicine, Necmettin Erbakan University, Konya, Turkiye; 2 Department of Medical Biochemistry, Konya City Hospital, University of Health Sciences, Konya, Turkiye; 3 Department of Otolaryngology, Faculty of Medicine, Necmettin Erbakan University, Konya, Turkiye; 4 Department of Radiation Oncology, Faculty of Medicine, Ankara Yıldırım Beyazıt University, Ankara, Turkiye; 5 Department of Medical Biochemistry, Faculty of Medicine, Necmettin Erbakan University, Konya, Turkiye

**Keywords:** Irradiation, melatonin, thymoquinone, antioxidant enzymes, radioprotection

## Abstract

**Background/aim:**

This study aimed to investigate thymoquinone (TQ), and melatonin’s radioprotective effects on liver, parotid gland, brain, and testis of rats which were exposed to total body irradiation (IR).

**Materials and methods:**

Thirty adult Wistar rats were randomly divided into four groups that are Group 1 (control group): total body IR only, Group 2: IR-Melatonin (10 mg/kg), Group 3: IR-TQ (10 mg/kg), and Group 4 (sham group): nothing. Total body IR dose was 6 Gy. Tissue samples were taken 90 min after IR. The measurements of malondialdehyde (MDA), glutathione peroxidase (GSH-Px), and superoxide dismutase (SOD) were performed in all groups.

**Results:**

In IR group, GSH-Px and SOD activities significantly decreased whereas MDA levels significantly increased when compared with the sham in all tissues. We recorded a significant decrease in MDA levels in IR-TQ group in liver and parotid gland of rats. Moreover, SOD did not change in IR-TQ group compared with IR only group.

**Conclusion:**

Melatonin, a powerful antioxidant, plays role in preventing oxidative stress. We revealed that premedication with TQ significantly inhibited the increase in MDA induced by IR in liver and parotid gland and protected the activities of SOD, an antioxidant enzyme, in all other tissues. It has been revealed that TQ has a potential effect preventing IR-induced damage as much as melatonin.

## 1. Introduction

Radiotherapy (RT), one of the main methods of cancer treatment, reduces the rate of recurrence and death. Ionizing radiation (IR) damage is used in RT and a process in which the excess energy is emitted by an unstable nucleus to achieve stability [1]. Failure to repair the damage caused by DNA double-strand breaks, the most common injury caused by IR, leads to cell death [2].

There is a close relationship between IR-induced free radicals and oxidative damage. The oxidative stress damages nucleic acids and induces protein and lipid peroxidation, thereby causes direct cellular injury. Since the most important free radicals are oxygen-containing they are frequently referred as reactive oxygen species (ROS) in biological systems [3].

Antioxidant system is a defense mechanism against ROS developed by cells and consists of enzymatic and nonenzymatic components [4]. Low-molecular-weight antioxidant compounds such as melatonin and glutathione (GSH) and several antioxidant enzymes such as glutathione peroxidase (GSH-Px) and superoxide dismutase (SOD) constitute the antioxidant system [5–7].

Malondialdehyde (MDA) is a marker of oxidative stress and arises from lipid peroxidation. It is also a well-characterized mutagen forming an important endogenous adduct, which is found in DNA, through reacting with deoxyguanosine. This highly reactive compound, MDA, has been used as the index of oxidative damage [8].

Sensitivity in surrounding normal tissues determines the IR dose that can be used for tumor. Therefore, strategies developed to improve IR aim to raise the influence on tumor or decline the influence on normal tissues [9]. The search for cytoprotective agents has been needed in order to reduce the toxicity of IR. The role of radioprotectors in treatment is limited as most of them have toxic effects [10,11].

Thymoquinone (TQ) is the major compound of volatile oil which originates from Nigella sativa seeds and has been used in folk medicine for centuries and it has been revealed that it has many biological functions, particularly antiinflammatory, antioxidant, and anticarcinogenic functions [12–16].

Melatonin, which is secreted by pineal gland, functions as a powerful endogenous free radical scavenger and antioxidant. This hormone has commonly been used as a protective agent for tissues against free radical injuries. Additionally, antiapoptotic effect of melatonin has been observed [17, 18].

The present study aimed to show that TQ has a radioprotective effect at least as much as melatonin on antioxidant and oxidant parameters in some radiation sensitive tissues such as liver, parotid gland, brain and testis of rats exposed to total-body IR.

## 2. Materials and methods

### 2.1. Animals and ethical guidelines

The study was conducted following the approval of the Ethics Committee from the Animal Care Committee at KONUDAM Experimental Medicine Research and Study Center (decision number: 073) and in accordance with the European Community guidelines and Helsinki and Tokyo Declarations. At the beginning of the experiment, thirty adult male Wistar rats weighing 200–250 g, 10–12 weeks old, were randomly divided into 6 animals in the sham group and 8 animals in the other groups. Rats were fed with standard rat pellets and water ad libitum and cages in which the rats were housed were in a laboratory without windows at a temperature of 20 to 23 °C and lighting controls (12 light/12 dark cycle). All procedures were conducted under sterile conditions.

### 2.2. In vivo experiments and groups

Rats were randomized into four treatment groups in the beginning of the experiment as follows:

Group 1 (control group) received IR and 0.9% physiological saline solution; melatonin at a dose of 10 mg/kg and IR were administered to Group 2 (IR + melatonin group); Group 3 (IR + TQ group) received 10 mg/kg TQ plus IR; and Group 4 (sham group) did not receive anything. The rats received IR under general anesthesia for immobilization before radiation exposure. Thirty min after intraperitoneal administration of melatonin and TQ, anesthesia with 50 mg/kg dose of Ketamine HCL was administered to all animals. Placed on a plexiglass tray, the animals were stabilized in supine position. Groups 1, 2, and 3 received total body irradiation with 6 Gy as a single fraction. The 6 MV photon beam energies (Linear Accelerator, Siemens, USA) were used for IR. There was a distance of 100 cm from source centre to skin in the source-axis distant technique. After IR was administered the rats were taken back to their cages. They received anesthesia in the same way after one and a half h. Submandibular salivary gland, liver, testis, and brain tissue were removed and rats were sacrificed. Tissues were taken into saline for biochemical analysis. The National Research Council’s guide for care and use of laboratory animals were followed during the experiments [19].

### 2.3. Biochemical analysis

Tissues were homogenized in phosphate buffer. The clear liquid lying above after the centrifugation of homogenate at 10,000 Χ g for 1 h to eliminate debris, supernatant was obtained. Tubes were kept at 4 °C during all the procedures and −80 °C during storage of samples until the study was performed.

### 2.4. Determination of malondialdehyde levels

MDA is an end product of unsaturated fatty acid peroxidation and a similar method described by Draper et al. was used for its spectrophotometrical determination. When heated in an acid medium in a water bath MDA can react with thiobarbituric acid (TBA) which forms thiobarbituric acid-reactive substances. Our method was based on the formation of MDA-TBA complex absorbed at 532 nm. The data obtained were given in nmol/mg protein [20].

### 2.5. Measurement of glutathione peroxidase and superoxide dismutase activities

In the study, tissue SOD and GSH-Px enzyme activities were measured respectively with RANSOD and RANSEL kits (Randox Laboratories Ltd., Crumlin, UK) by colorimetric assay on the Architect c16000 chemistry analyzer (Abbott Diagnostics, USA). GSH-Px and SOD activity values were calculated as mU/mg protein.

### 2.6. Statistical analysis

Data were statistically analyzed with the SPSS 22.0 for Windows (SPSS Inc., Chicago, IL) software program. The results were statistically compared with One-way analysis of variance (ANOVA) test. P values less than 0.05 were statistically significant. Quantitative data were expressed as mean ± standard deviation (SD).

## 3. Results

Radiation-only group had significantly higher MDA levels in all of the tissues compared with the sham group. As shown in Table 1, a significant reduction was recorded in MDA values of the salivary gland and liver in the IR-TQ group and MDA values of the brain and testis in the IR-melatonin treatment group (p < 0.05).

A decrease was observed in SOD and GSH-Px activities of IR treatment group compared to the sham group in all tissues (p < 0.05). Other groups receiving IR (Group 2 and 3) had significantly lower GSH-Px activity compared to the sham group (Table 2).

No statistically significant increase was observed in SOD activity in IR-TQ group while liver and testis SOD activity was significantly higher in IR-melatonin group compared with the radiation-only group. SOD activities of the sham group were significantly higher in all tissues compared to the control group (p > 0.05) (Table 3) (Figure).

## 4. Discussion

IR provides treatment for various indications in cancer; however, it has also harmful effects caused by the formation of free radicals. Presence of any agent that can reduce toxicity of IR on normal cells would be very valuable in decreasing the side effects [21]. This study investigated the radioprotective effects of melatonin and TQ on the damage induced by radiation when the rats received total-body IR at a single-dose.

The study characterized IR-mediated oxidative damage in liver, parotid gland, brain, and testis of rats. Concentration of an oxidative stress parameter, MDA and antioxidant status on the basis of some antioxidant parameters such as SOD and GSH-Px activities estimate the oxidative stress. Baseline parameters of IR groups were compared with those of sham and control groups. Although a significant increase was recorded in MDA of IR-only group compared to sham group, a significant decline was recorded in SOD and GSH-Px activities in all tissues. Our results demonstrated that lipid peroxidation significantly increased in liver, parotid gland, brain, and testis of the rats receiving total-body IR at a single dose. Additionally, IR resulted in a significant increase in oxidant levels and decline in antioxidant levels in liver, parotid gland, brain, and testis of the rats. These results were compliant with those of previous studies performed on different tissues [22–25].

Radiotherapy (RT) is a treatment method to destroy cancer cells while damaging normal cells as little as possible. On the other hand, normal tissues cannot always be kept outside the field of treatment and side effects and complications due to IR cannot always be avoided. Presence of any agent that can reduce the toxicity of IR on normal cells would be very valuable in decreasing the side effects [26].

Melatonin, a well-known powerful antioxidant, has potential antioxidative and radioprotective properties [27]. Melatonin is thought to be extremely strong free radical scavenger [28]. High lipid solubility of melatonin enables it to pass through the cell membrane preventing the initiation of events that result in lipid peroxidation [29]. Tahamtan et al. [30] suggested that melatonin decreased MDA levels elevated with IR in lung tissue of rats and that lung injuries induced by ionizing radiation could be reduced with melatonin treatment. In agreement with this result, we found that melatonin decreased MDA levels in liver, parotid gland, brain, and testis but this decrease was statistically significant only in brain and testis. In addition to melatonin’s direct action, it can increase antioxidant enzyme activities such as SOD or GSH-Px activity [31]. The melatonin group had significantly higher levels of both liver and testis SOD compared with radiation-only group, which is consistent with findings on the antioxidant effects of melatonin in literature.

Providing an effective radioprotection is of great medical importance. Plant compounds are natural radioprotectors and can protect normal cells from RT-induced damage. Tumor killing has been enhanced using the herbals and dietary modulators together with IR [32]. One of the major mechanisms offering radioprotection with the use of plants and phytochemicals is antioxidant activity [33]. Interestingly, our results showed that premedication with TQ significantly prevented the increase in MDA levels induced by IR, suggesting that TQ may have the potential protective effect against the IR-induced damage. In addition, SOD activities were significantly decreased only in liver, parotid gland, brain and testicles of IR group rats; however, no statistically significant reduction was observed in the IR + TQ treatment group compared to the sham and IR-only groups. The antioxidant effect of TQ has also been confirmed by our results, which showed that TQ induces the complete reversal of the IR-induced increase in MDA levels and protects the activities of antioxidant enzymes in salivary glands compared to the normal control values. TQ has been exposed to many pharmacological investigations in recent years. Many studies have been reported that premedication with TQ protects organs against oxidative damage induced by a variety of free radical generating agents, including carbon tetrachloride and cisplatin, and have the antioxidant effects of the TQ and a free radical scavenging activity [34,35]. X-ray exposure leads to significant changes in cellular morphological and biochemical conditions. It has been demonstrated by a study this adverse effects that develop as a result of IR treatment will be significantly reduced with TQ application before radiation, and that the in vivo cardioprotective effect of TQ may offer a new therapeutic approach [1].

Radioprotectors ideally are expected to have selectivity for normal tissues, but not for tumor tissues from the effect of RT. However, the current study does not provide any data on comparing the effect of TQ on tumor and normal tissues. This is a limitation of our study.

As a result, according to the results of our study investigating how TQ affects oxidant-antioxidant system in liver, parotid gland, brain, and testes of irradiated rats; the increase in IR-induced MDA levels in liver and parotid gland was prevented by premedication with TQ. In addition, TQ did not change SOD activities in IR+TQ group in all tissues, which suggests that TQ has a potential protective effect on radiation injury. When administered before IR through eliminating of radiation-induced free radicals, TQ has a potential to decrease the effects of radiation.

However, further experimental studies are needed to explain the molecular mechanism of TQ protective effects and also to uncover if TQ can have any selectivity for normal tissues, but not for tumor tissues, from the effect of RT.

## Figures and Tables

**Figure:**
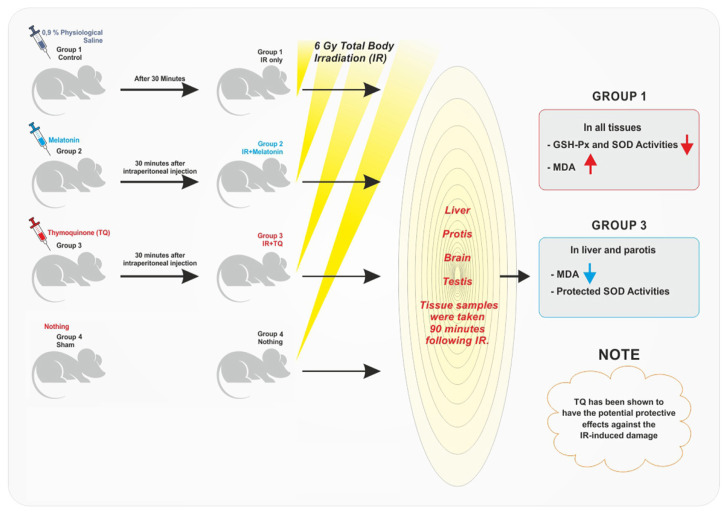
Graphical summary of the study procedure.

**Table 1:** Mean ± standard deviation of malondialdehyde (nmol/mg protein) in all groups.

	Liver	Parotid gland	Brain	Testis
**Group 1**, Control (radiation only)	3.73 ± 1.01^d^,^c^	14.62 ± 15.31^d^,^c^	21.88 ± 5.59^d^,^b^	15.23 ± 12.39^d^,^b^
**Group 2** (10 mg kg^−1^ melatonin + radiation)	3.09 ± 0.86	7.04 ± 5.76	12.75 ± 5.12^a^	7.87 ± 3.78^a^
**Group 3** (10 mg kg^−1^ thymoquinone + radiation)	2.08 ± 0.57^a^	4.28 ± 2.48^a^	16.96 ± 5.42	8.86 ± 1.82
**Group 4**, Sham	2.26 ± 0.39^a^	3.06 ± 1.43^a^	15.94 ± 5.05^a^	7.84 ± 4.98^a^

a p < 0.05 compared to radiation-only group.

b p < 0.05 compared to melatonin + radiation group.

c p < 0.05 compared to thymoquinone + radiation group.

d p < 0.05 compared to sham group.

**Table 2:** Glutathione peroxidase activity (mU/mg protein) in each study group.

	Liver	Parotid gland	Brain	Testis
**Group 1**, Control (radiation only)	601.13 ± 32.22^d^	223.94 ± 77.29^d^	202.67 ± 45.24^d^	161.75 ± 35.18^d^
**Group 2** (10 mg kg^−1^ melatonin + radiation)	610.40 ± 40.66^d^	260.94 ± 113.68^d^	225.63 ± 48.50^d^	178.58 ± 44.21^d^
**Group 3** (10 mg kg^−1^ thymoquinone + radiation)	611.55 ± 135.19^d^	324.32 ± 105.79^d^	234.14 ± 45.92^d^	180.94 ± 26.20^d^
**Group 4**, Sham	812.50 ± 162.18^a^,^b^,^c^	673.93 ± 496.07^a^,^b^,^c^	321.21 ± 46.62^a^,^b^,^c^	235.39 ± 29.23^a^,^b^,^c^

a p < 0.05 compared to radiation-only group.

b p < 0.05 compared to melatonin + radiation group.

c p < 0.05 compared to thymoquinone + radiation group.

d p < 0.05 compared to sham group.

**Table 3:** Mean ± standard deviation of superoxide dismutase activity (mU/mg protein) measured in the liver, parotid gland, brain, and testis of rats.

	Liver	Parotid gland	Brain	Testis
**Group 1**, Control (radiation only)	22.07 ± 2.56^b^,^d^	35.53 ± 21.65^d^	45.14 ± 4.25^d^	29.27 ± 2.37^d^,^b^
**Group 2** (10 mg kg^−1^ melatonin + radiation)	33.03 ± 17.79^a^	45.21 ± 15.58	48.85 ± 2.03^d^	34.89 ± 4.52^a^
**Group 3** (10 mg kg^−1^ thymoquinone + radiation)	26.05 ± 2.49	24.99 ± 4.31	47.11 ± 2.77	31.8 ± 4.11
**Group 4**, Sham	33.33 ± 3.77^a^	86.73 ± 66.18^a^	56.21 ± 7.29^a^,^b^	36.6 ± 2.49^a^

a p < 0.05 compared to radiation-only group.

b p < 0.05 compared to melatonin + radiation group.

c p < 0.05 compared to thymoquinone + radiation group.

d p < 0.05 compared to sham group.
